# LAT, HOXD3 and NFE2L3 identified as novel DNA methylation-driven genes and prognostic markers in human clear cell renal cell carcinoma by integrative bioinformatics approaches

**DOI:** 10.7150/jca.35641

**Published:** 2019-10-22

**Authors:** Jie Wang, Hu Zhao, Huiyue Dong, Ling Zhu, Shuiliang Wang, Ping Wang, Qun Ren, Hehuan Zhu, Junqiu Chen, Zhijie Lin, Yuanhang Cheng, Benjiang Qian, Yi Zhang, Ruxue Jia, Weizheng Wu, Jun Lu, Jianming Tan

**Affiliations:** 1 Fujian Provincial Key Laboratory of Transplant Biology, Department of Urology, Dongfang Hospital (900 Hospital of the Joint Logistics Team), Xiamen University, Fuzhou, 350025, Fujian, P.R. China.; 2 Fujian Provincial Key Laboratory of Transplant Biology, Department of Urology, Fuzhou General Clinical College, Fujian Medical University, Fuzhou, 350025, Fujian, P.R. China.; 3 Fujian Hongyi Health Institute, Fuzhou, 350025, Fujian, P.R. China.

**Keywords:** methylation-driven, expression, clear cell renal cell carcinoma, CpG, bioinformatics

## Abstract

**Background:** Abnormal DNA methylation of is one of the important mechanisms leading to tumor pathogenesis. The purpose of this study was to explore differentially methylated genes that may drive the development of renal clear cell carcinoma through a comprehensive analysis of the TCGA database. **Materials and methods:** Methylation data and RNA-seq data for clear cell renal cell carcinoma were downloaded from The Cancer Genome Atlas (TCGA). Differentially methylated genes and the differential genes associated with survival were then screened by MethylMix R package and univariate Cox proportional-hazards model, respectively. Their common genes were then intersected and obtained for further analysis. Correlation of gene expression and methylation levels, gene set enrichment analysis (GSEA) enrichments, survival curve, and ROC curve plotting for DNA methylation-driven genes were finally performed. The methylation alterations of the three genes were validated via two GEO datasets (GSE70303 and GSE113501), and the genes expression level was verified through two GEO datasets (GSE6344 and GSE53757). **Results:** Three novel DNA methylation-driven genes *LAT*, *HOXD3* and *NFE2L3* were identified in clear cell renal cell carcinoma. Expression analysis further revealed that hypomethylation levels of *LAT* and *NFE2L3* showed higher gene expression levels, while *HOXD3* exhibited opposite methylation-expression pattern. The CpG sites of *LAT* (cg16462073), *HOXD3* (cg24000528) and *NFE2L3* (cg16882373) that may affect respective gene expressions were also identified. For the survival analysis, we found that hypomethylation and over-expression of LAT and NFE2L3 were correlated with poor survival, while hypermethylation and low-expression HOXD3 was correlated with poor survival of clear cell renal cell carcinoma patients. In addition, GSEA KEGG analysis and biological processes of these genes were also enriched for functional analysis. Kaplan-Meier survival and ROC analyses of these genes showed an average risk score of 0.9140593, AUC = 0.692, which suggested a good clinical application value. Finally, the opposite methylation-expression pattern of these three genes were verified in GEO datasets. **Conclusions:** In this study, we successfully exhibited the potential DNA methylation-driven genes *LAT*, *HOXD3*, and *NFE2L3* involved in clear cell renal cell carcinoma. Moreover, gene functions and prognostic risk models were also elucidated, which facilitated the expansion of the current study on the role of methylation in the pathology process of clear cell renal cell carcinoma.

## Introduction

Renal cell carcinoma (RCC) is a tumor originating from renal tubular epithelial cells, which is the most common type of renal cancer 1. According to the data of the GLOBOCAN 2018, 403262 (2.2% of the total) new cases of RCC patients were diagnosed, and 175,098 (1.8% of the total) patients died of this disease 2. Clear cell renal cell carcinoma (ccRCC) is the most common pathological subtype of RCC, which accounts for 75-80% of RCC 3, 4. Nowadays, nephrectomy is still the most effective treatment approach for localized ccRCC. Conventional chemoradiotherapies are good options for several malignant cancer types, however, for ccRCC is a highly angiogenic cancer by secreting vascular endothelial cell growth factor (VEGF), it is really not so sensitive for chemoradiotherapies treatment 5, 6. Standard treatments for metastatic ccRCC include high doses of interleukin 2 (HDIL-2) and interferon alpha (IFNα) 3, 7, and the first-line targeted drugs including Sorafenib, Suntinib 8, 9. Although these therapies improve the survival of ccRCC patients to a certain extent, the toxicities, side effects, and tolerance of these drugs are still a challenge during ccRCC treatment process. In order to further discover effective therapeutic targets, it is particularly urgent to explore new drug targets or biomarkers for ccRCC treatment.

DNA methylation is the process of the covalent addition of the methyl group at the 5-carbon of the cytosine ring of CpG dinucleotides under the action of DNA methyltransferase (DNMT). In the different stages of cancer development, abnormal hypermethylation of CpG islands in gene promoter regions will occur, which results in transcriptional silencing and abnormal expression of multiple key genes 10. Alterations in DNA methylation including hypermethylation of tumor suppressor genes and hypomethylation of oncogenes have been widely recognized as important regulators in cancer development 11. Yusuke Sato et al. found that DNA methylation was correlated with the clinical progressions of ccRCC 12. Shenoy et al. also discovered that the low expression of numerous tumor suppressor genes aroused by hypermethylation of enhancers and promoter regions leads to enhanced tumor cell biological activities in ccRCC 13. However, these studies did not systematically screen for DNA methylation-driven genes in the progression of ccRCC. The Cancer Genome Atlas (TCGA) 14 is a commonly used public database with a large amount of data that facilitates relevant disease and prognostic analysis. Therefore, the specific objective of this study was to mine and screen DNA methylation-driven genes for ccRCCs through the TCGA KIRC database.

## Materials and Methods

### Data collection and preprocessing

**Figure 1:**
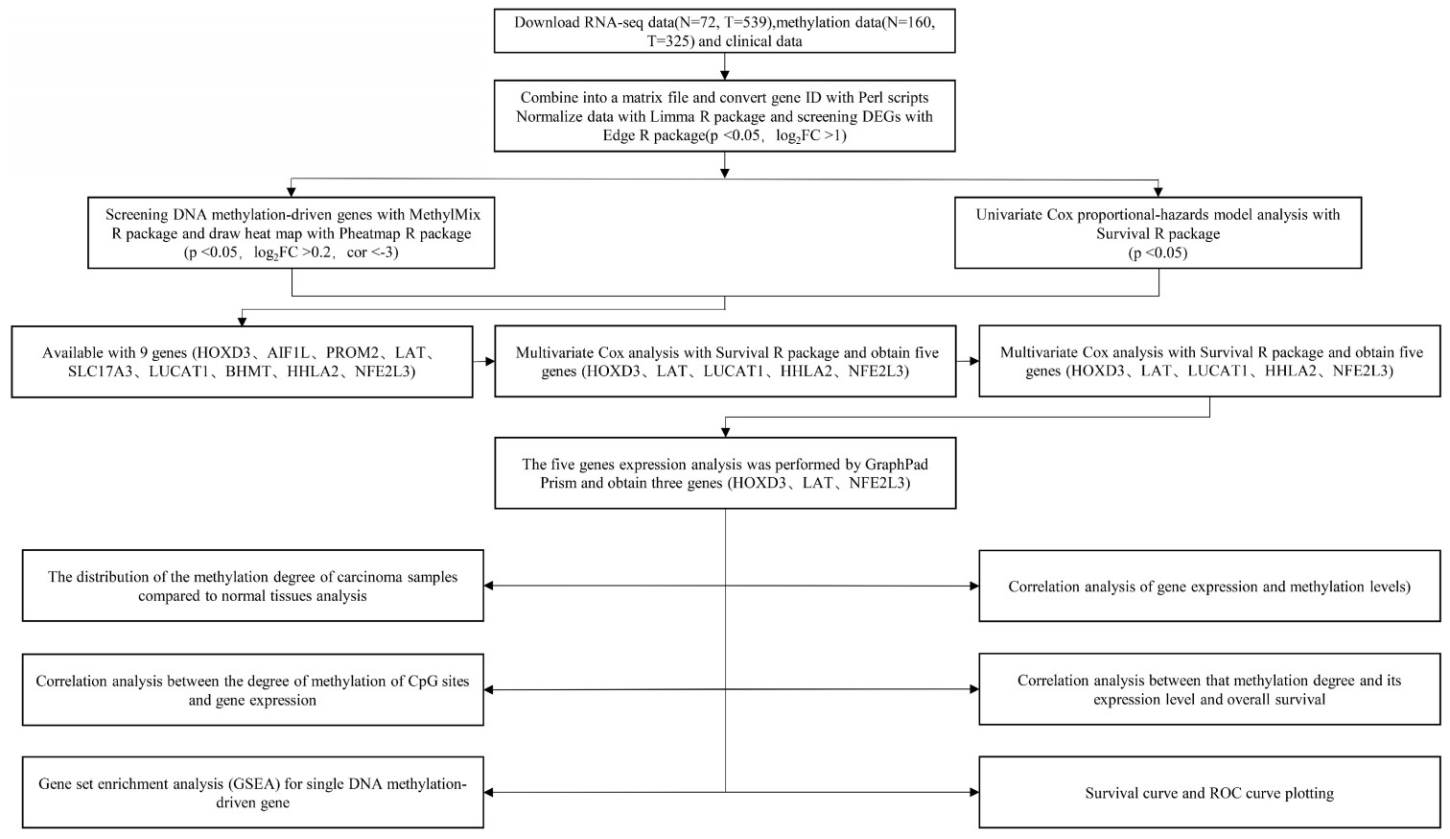
Technical roadmap for this study designed.

The technical roadmap was shown in **Figure 1**. Firstly, RNA-seq data (N = 72, T = 539), DNA methylation data (N = 160, T = 325) and clinical data of the original ccRCC patients were downloaded from the TCGA database. Separately the original RNA-seq data and methylation data files were merged into single matrix file using the merge script of Perl 5.24.3 language. Similarly, the survival time and survival state of the samples were extracted into the matrix file by the Perl language. Then, the Ensemble ids of gene names were converted into gene symbols by means of the Perl 5.24.3 language script. The matrix was then normalized using the Limma R package. Finally, the differentially expressed genes (DEGs) were screened using the Edge R package. This study was strictly in accordance with the published guidelines issued by TCGA.

### DNA methylation-driven genes screening

The methylation-driven genes in ccRCC were screened with the use of the MethylMix R package (*p* < 0.05, Log_2_FC > 0.2). Differential distribution picture of these genes was drawn by employment of Pheatmap R package. Through two key functionalities of the MethylMix R package: creating a MethylMix model map for each gene and mapping a linear regression model between DNA methylation and gene expression 15, distribution of methylation degree in ccRCC samples and its correlation with respective gene expressions were performed.

### Analysis of univariate and multivariate Cox regression with DEGs of ccRCC

Univariate Cox regression analysis with DEGs of ccRCC was performed with the use of the Survival R package. Subsequently, the outcome of univariate Cox analysis minings were conducted by means of *p* < 0.001 and 0.64 < HR < 1.44.

### Relative expressions of DNA methylation-driven genes in ccRCC

The expression map of novel DNA methylation-driven genes in normal and ccRCC samples was plotted with the use of GraphPad Prism software. The data was standardized log2-gene expression values.

### Correlation analysis between CpG site methylation degrees and gene expressions

Firstly, the CpG site methylation degree matrix was obtained using the Perl language script. Then, R software was employed to combine the methylation degrees of the CpG site with the gene expression matrix to form a new matrix file, and the correlation between them were further analyzed. The CpG site information was referenced by the Illumina Human Methylation 450 BeadChip. The Wanderer, a web analytics platform to reveal the position of the CpG sites on the chromosome, was finally employed to uncover the methylation degrees of the CpG sites in the ccRCC patients 16.

### Gene set enrichment analysis (GSEA) for every DNA methylation-driven gene

In the cancer group, we performed GSEA analysis for every novel DNA methylation-driven gene. Based on the median expressions of the DNA methylation-driven genes in the 539 renal clear cell carcinoma samples, we divided them into high and low expression groups. After normalization of gene expressions data, Gene Ontology (GO) and Kyoto Encyclopedia of Genes and Genomes (KEGG) pathway analysis were performed by employment of GSEA software. Gene sets related to biosignaling found on MSIGDB (Molecular Signature Database) could be found on the GSEA website as reference gene sets. According to the default weighted enrichment statistics methods, the process was repeated 1,000 times for each analysis. Statistically significant pathways and biological processes were screened based on *p* < 0.05, FDR < 0.25, and *p* < 0.05, FDR < 0.05, respectively. After sorting according to the NES value, the top gene sets were selected for each enrichment result for study.

### Combination analysis of methylation levels, gene expressions and survive rate

Firstly, three types of data (survival times, methylation degrees and gene expressions) were combined into one matrix file by using the Hash R package. Then, the Survival R package was used for the joint analysis of these data.

### Survival curve and ROC curve plotting

According to the survival scores obtained from the multivariate Cox regression analysis and the DNA methylation-driven gene expression values of each sample, patients were divided into high-risk group and low-risk group by calculating the median value of the risk score of each sample. The Kaplan-Meier survival curves of high-risk and low-risk groups were plotted by using the Survival R package. The ROC curve was then plotted with the use of the survival ROC R package, which could predict the accuracy of the Cox proportional-hazards model. Heat map was Plotted with the employment of the pheatmap R package.

### Analysis of the methylation and expression pattern of three DNA methylation-driven genes in Gene Expression Omnibus (GEO) datasets

GSE70303 and GSE113501 17, 18 were chose to verified the three DNA methylation-driven genes methylation-patterns. GSE70303 includes 6 normal renal samples and 6 ccRCC samples, GSE113501 includes 12 normal samples and 132 ccRCC samples. Both of these datasets were the results of Illumina 450k Human Methylation BeadChip detection. GSE6344 and GSE53757 were used to validate the comparison expression of DNA methylation-driven genes between normal tissues and ccRCC tissues 19, 20. GSE6344 includes 10 normal renal samples and 10 ccRCC samples. GSE53757 includes 72 normal samples and 72 ccRCC samples. To improve the number of samples, two of datasets were integrated respectively. Finally, methylation matrix contains 18 normal tissues and 138 ccRCC tissues; expression matrix contains 82 normal tissues and 82 ccRCC tissues. Sva R package was used for rejecting the batch effect between two of the datasets 21. Limma R package was used to normalize data. For gene methylation analysis, a total beta value for each gene was obtained via computing average methylation value for all CpG sites related to a gene in each sample.

### Statistical analysis

All data were conducted at least three times and represent data from at least three separate experiments. Two-tailed Student's t-test was utilized for significance of differences between groups. Statistical was processed via R software 3.4.4 and Graph-Pad Prism 6.0 (GraphPad Software, Inc., La Jolla, CA, USA). Probability values of *p* < 0.05 was considered as significant difference.

## Results

### Identification of DNA methylation-driven genes in TCGA KIRC samples

**Figure 2:**
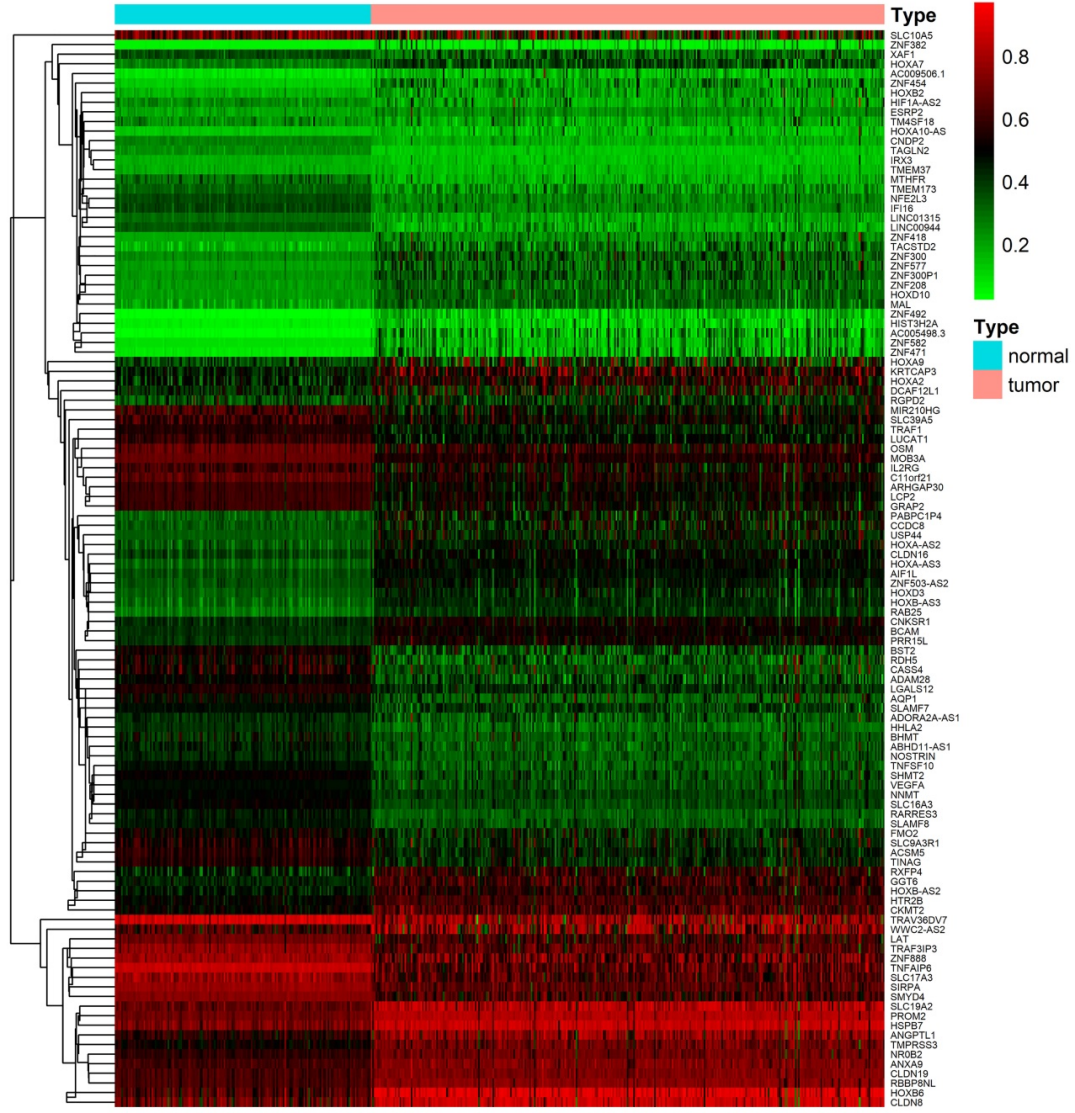
Heat map of differential methylation genes. Bidirectional hierarchical clustering of differentially methylated genes in renal clear cell carcinoma and adjacent tissues. Red in the figure indicates that the gene of hypermethylation in the sample and green indicates that the gene of hypomethylation in the sample.

After downloading and preprocessing of methylation data, we analyzed the DNA methylation-driven genes of TCGA KIRC with the MethylMix R package, using *p* < 0.05, Log_2_FC > 0.2 and cor < -3 as the cutoff criterion. We screened 56 hypermethylated genes and 56 hypomethylated genes in TCGA KIRC samples compared to normal adjacent samples (**Figure 2**,**Table S1**).

### Combination analysis of univariate and multivariate Cox regression with DNA methylation-driven genes

**Figure 3:**
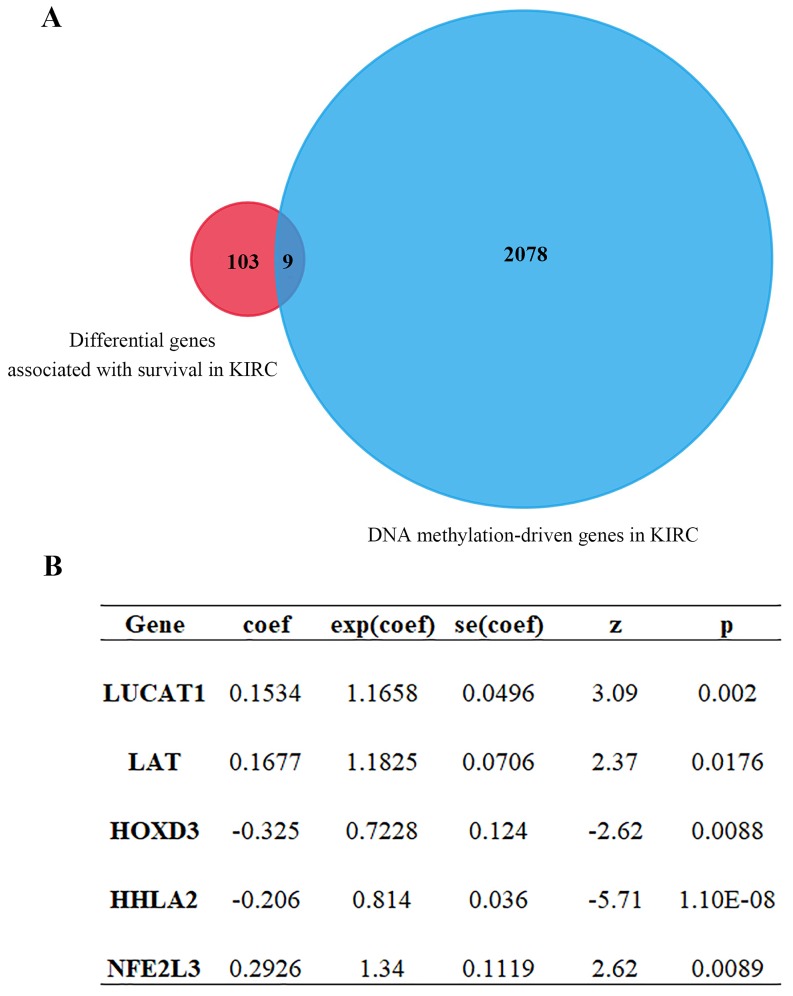
Cox proportional-hazards model analysis and MethylMix R package analysis. (A) Venn diagram showing intersection genes expression between gene set 1 and gene set 2 (9 genes). Gene set 1 is differential genes associated with survival; Gene set 2 is DNA methylation-driven genes. (B) 5 optimal gene models obtained by multivariate Cox analysis.

Univariate Cox regression analysis of ccRCC DEGs was performed by using the Survival R package, the screening condition was *p* < 0.001 and 0.64 < HR < 1.44. 2087 differential genes associated with survival were obtained (**Table S1**). After intersection for differential genes associated with survival and DNA methylation-driven genes, 9 common genes were further yielded (**Figure 3A**,** Table 1**). After that, multivariate Cox proportional-hazards model analysis of 9 previous common genes were executed and 5 optimal genes including HOXD3, LAT, LUCAT1, HHLA2 and NFE2L3 were obtained (**Figure 3B**). Risk score = LUCAT1 (gene expression level) * 0.1534 + LAT * 0.1677 + HOXD3 * (-0.3247) + HHLA2 * (-0.2057) + NFE2L3 * 0.2926.

### Correlation analysis between gene expressions and methylation degrees of novel DNA methylation-driven genes in ccRCC samples

**Figure 4:**
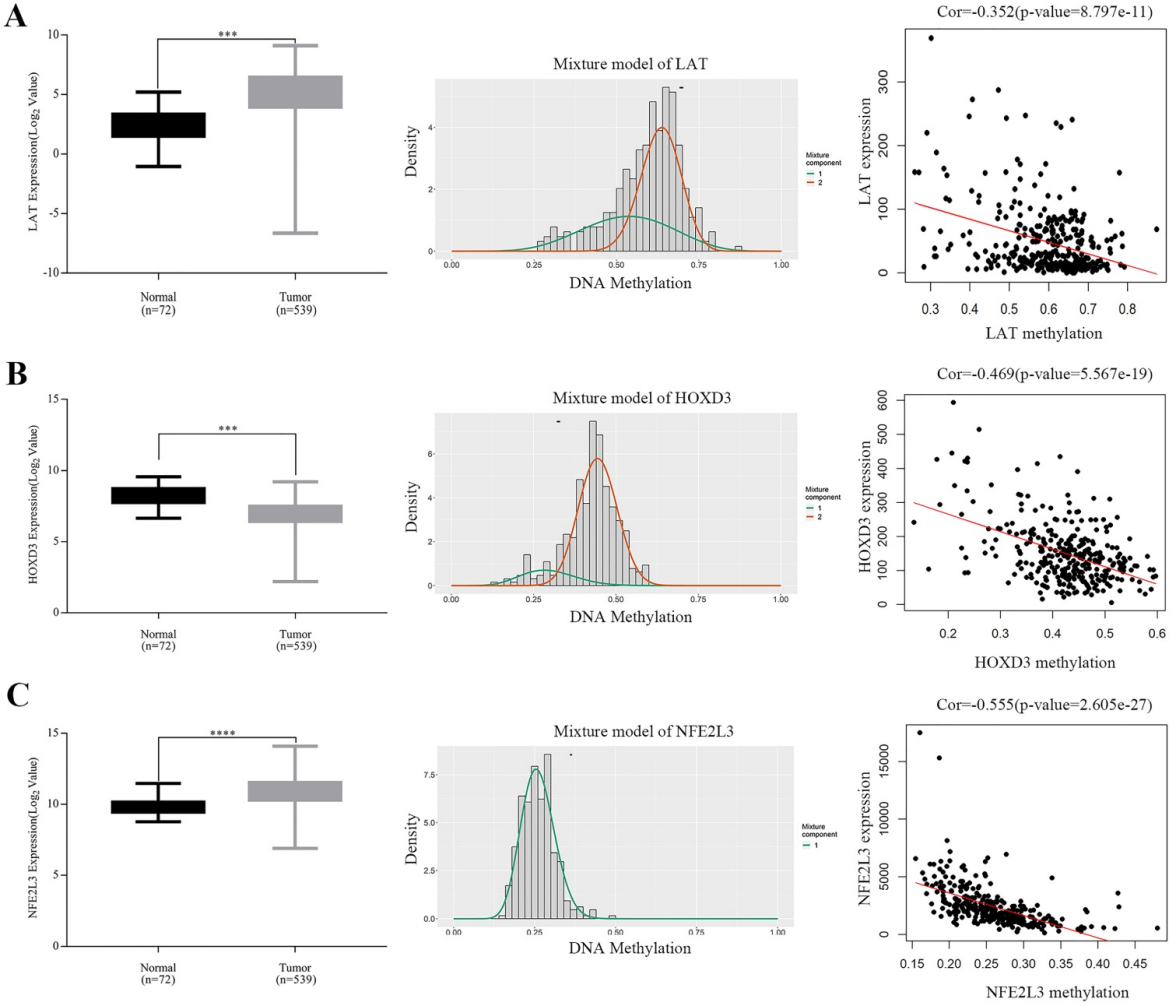
Boxplot showing relative expression of candidate DNA methylation-driven genes in normal and ccRCC samples A-C (left). The statistical analysis was performed by GraphPad Prism 7 (Two-tailed p value was determined by paired t-test, ***, *p* < 0.0001). Bars represent the Mean+SEM. Distribution chart of methylaption degree of candidate DNA methylation-driven genes A-C (middle). The abscissa represents the degree of methylation, the ordinate represents the number of methylation samples, the histogram shows the methylation distribution of tumor group, and the peak of the curve represents the average level of methylation distribution in the cancer group. The bold horizontal line represents the average level of methylation distribution in the normal group. A-C (right) showing Correlation analysis between methylation level and gene expression. The abscissa represents the beta value of the gene methylation degree, and the ordinate represents the expression of the gene. Cor is the correlation coefficient and P-value is the test value of correlation.

Expressions of five potential DNA methylation-driven genes were performed and it was found that only LAT, HOXD3 and NFE2L3, not LUCAT1 and HHLA2, were significantly up-regulated or downregulated in ccRCC samples compared with normal tissues, as shown in **Figure 4A-C** (left panel), **Figure S1A-B**. Methylation degree distributions of LAT, HOXD3 and NFE2L3 were plotted by using the MethylMix R package. As **Figure 4A-C** (middle panel) showed that LAT and NFE2L3 exhibited a hypomethylation state in the ccRCC cancer group, while HOXD3 exhibited a hypermethylation state in the ccRCC cancer group. More specific data was found in **Table 2**. In addition, correlation between methylation levels and gene expressions of LAT, HOXD3 and NFE2L3 was also analyzed by the MethylMix R package. Result showed that all methylation degrees of LAT, HOXD3 and NFE2L3 were negatively correlated with respective expressions in ccRCC,** Figure 4A-C** (right panel). We also mapped the specific CpG sites of these genes in ccRCC samples. As **Figure S2A-C** (left panel) showed that several CpG sites were widely distributed in LAT, HOXD3 and NFE2L3 genes. Combining the positional information of the CpG sites on the chromosome on the Illumina HumanMethylation450 BeadChip and correlation coefficient scores, we selected the following CpG sites for further correlation expression analysis: LAT (cg16462073), HOXD3 (cg24000528) and NFE2L3 (cg16882373) (**Table 4**). We found that the methylation degrees of CpG sites were negatively correlated with the expression of the respective genes (**Figure S2A-C**, right panel).

### GSEA KEGG and GO analysis for every novel DNA methylation-driven gene

**Figure 5:**
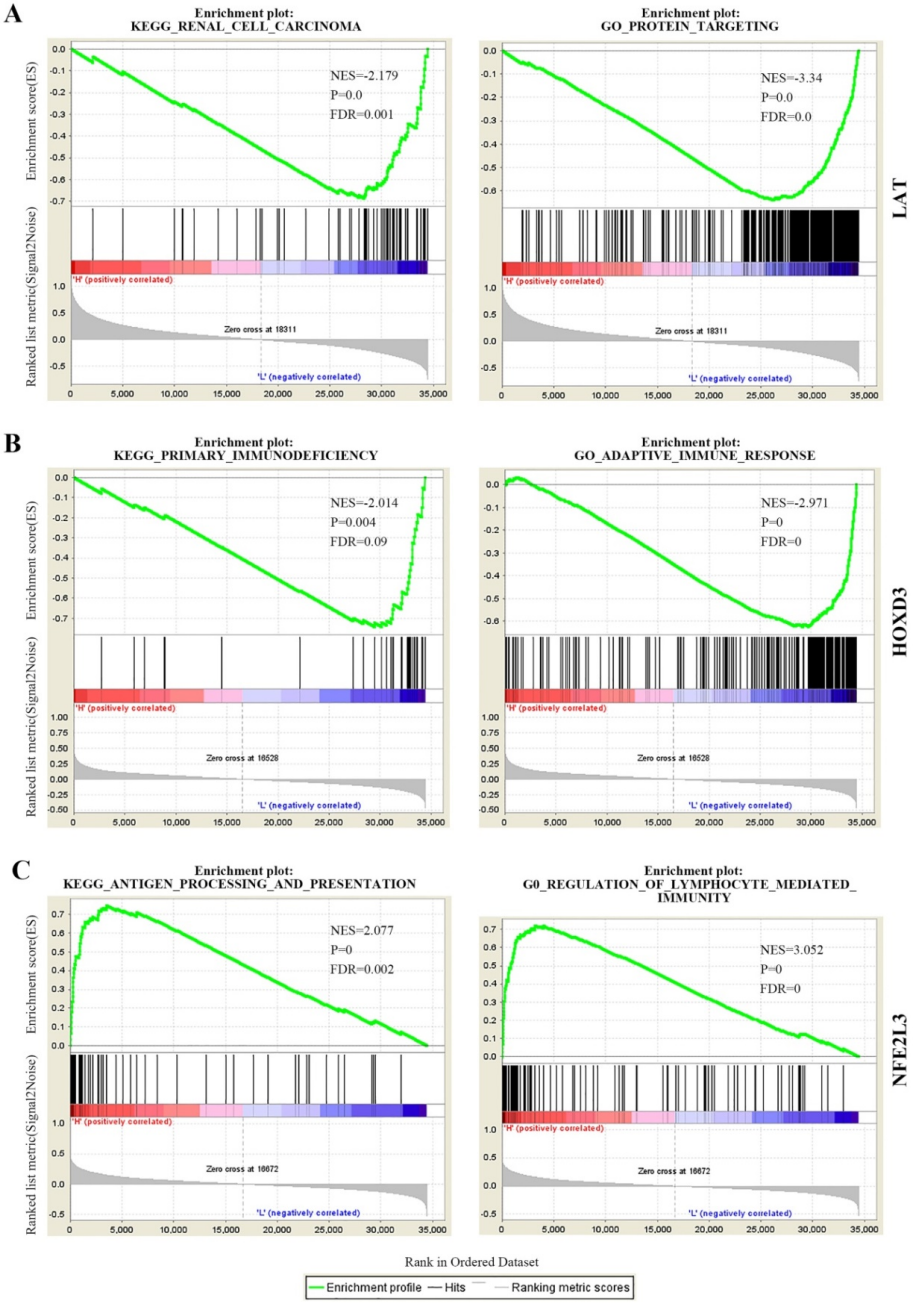
The partial KEGG pathways and GO terms were shown via the use of GSEA. (A) KEGG pathway and GO (biological process) of LAT. (B) KEGG pathway and GO (biological process) of HOXD3. (C) KEGG pathway and GO (biological process) of NFE2L3. KEGG, Kyoto Encyclopedia of Genes and Genomes; GO, Gene Ontology; NES, normalized enrichment score; FDR, false discovery rate.

To determine the biological function of DNA methylation-driven genes, both GO and KEGG pathway analysis were performed with the use of GSEA. The results of the KEGG pathway analysis showed that ubiquitin mediated proteolysis, renal cell carcinoma, ErbB signaling pathway, *et al* were significantly top-enriched for *LAT* gene; primary immunodeficiency, cytokine-cytokine receptor interaction, homologous recombination, *et al* were significantly enriched for *HOXD3*; antigen processing and presentation, cell adhesion molecules CAMS, *et al* were significantly enriched for* NFE2L3* (**Figure 5** left panel, **Table S2**). After that, GO biological processes analysis further revealed that protein targeting, translational initiation, amid biosynthetic process, *et al* were significantly enriched for *LAT*; adaptive immune response, positive regulation of interferon-gamma production, *et al* were significantly enriched for *HOXD3*; regulation of lymphocyte mediated immunity, positive regulation of cell-cell adhesion, *et al* were significantly enriched for *NFE2L3* (**Figure 5** right panel, **Table S3**). The above KEGG and GO biological processes analysis results can help us further understand the functions of DNA methylation-driven genes in ccRCC.

### Joint survival analysis of methylation and expression for DNA methylation-driven genes

**Figure 6:**
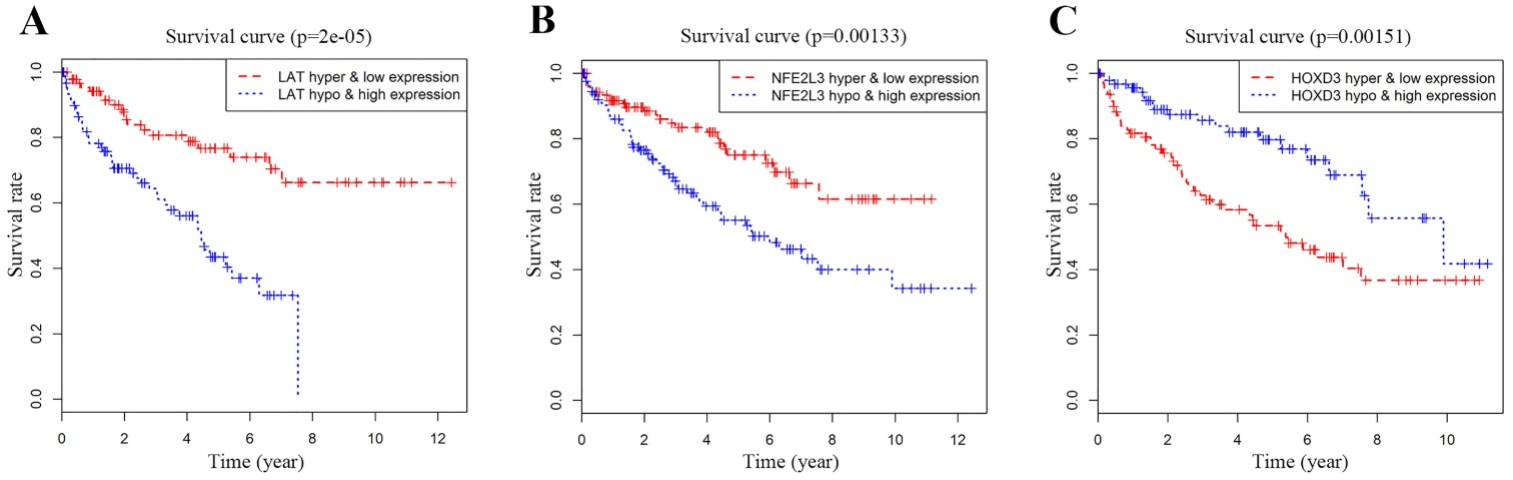
Kaplan-Meier survival analysis displaying correlation between that the Methylation degree of candidate DNA methylation-driven genes and their expression levels and overall survival (A-C). The abscissa indicates the overall survival time of the patient in years, ordinate denotes the survival rate.

Joint Prognostic survival analysis of DNA methylation-driven genes was performed by the surviving R package. We found that the combination of methylation and expression of the genes *LAT*, *NFE2L3*, and *HOXD3* had a significant correlation with the prognosis of the ccRCC patients. The hyper-methylation low-expression 5-year survival rate of *LAT* and *NFE2L3* were higher, while the hypo-methylation high-expression 5-year survival rate of the gene* HOXD3* was higher (**Figure 6**).

### Survival curve and ROC curve plotting for multivariate Cox proportional-hazards model analysis

**Figure 7:**
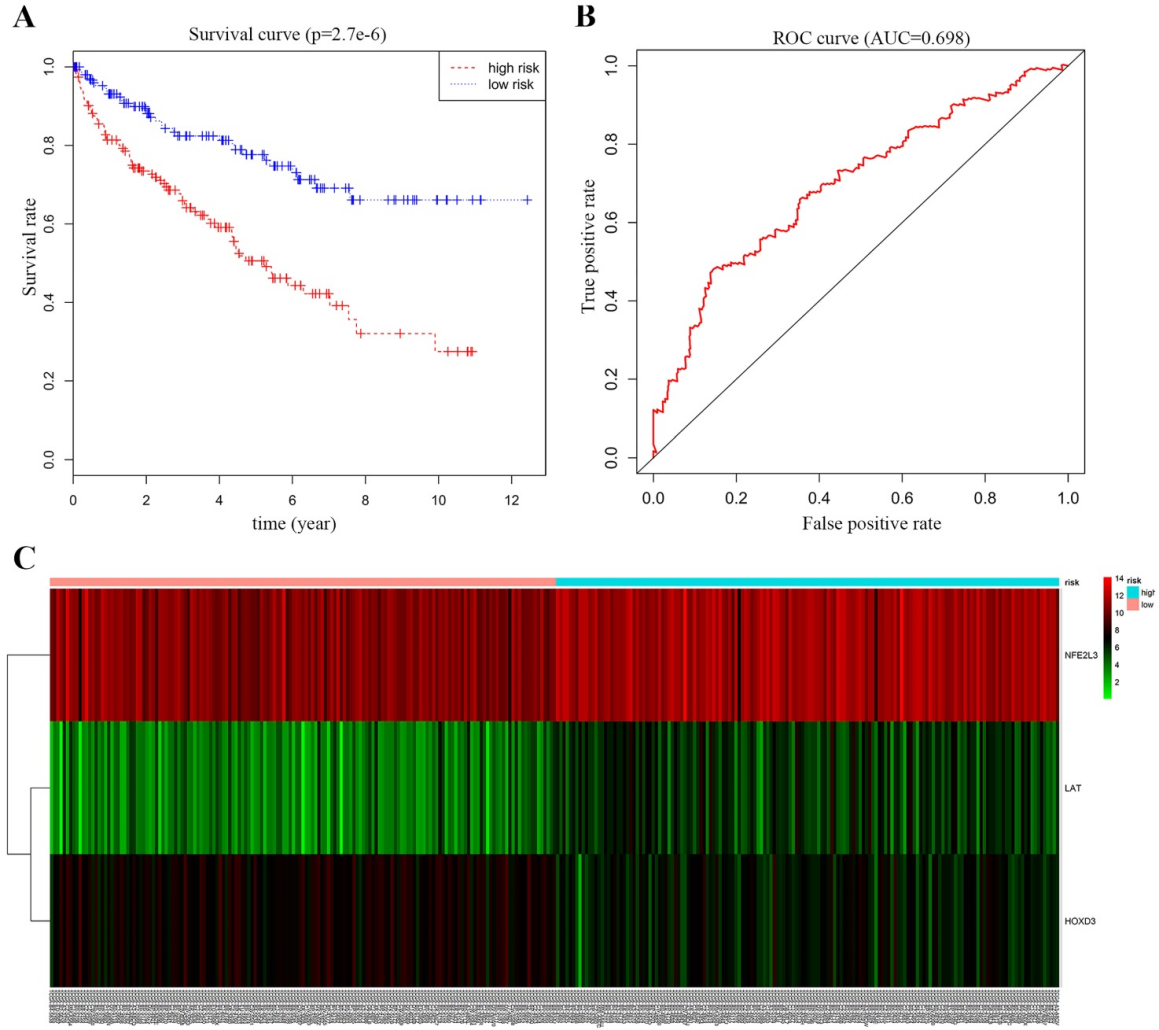
Survival curve and ROC curve plotting. (A) Kaplan-Meier statistical analyses of survival curves of the 539 patients with ccRCC according to candidate DNA methylation-driven genes expression. Kaplan-Meier survival curves were compared using log-rank tests. *p* < 0.001. (B) ROC curve demonstrates that our model can well predict ccRCC patient survival according to AUC = 0.698. (C) Heat map of high and low-risk group. Red: indicates a highly expressed gene; green: indicates a low expressed gene. From left to right of the abscissa, the risk score of the sample is getting higher and higher.

We performed multivariate Cox proportional-hazards model analysis for the obtained ccRCC methylation-driven genes with optimal risk model previously described. Risk score = LAT (gene expression level) * 0.2693 + HOXD3* (-0.3462) + NFE2L3 * 0.2414 as shown in **Table 5**. The results showed that the assessment model constructed by three genes (*LAT*, *NFE2L3*, and *HOXD3*) can be used as an independent indicator for predicting the prognosis of ccRCC. With the median value as the cut-off value, 159 patients with a risk score lower than the median value belonged to the low-risk group, and 158 patients with a risk score greater than the median value belonged to the high-risk group. Kaplan-Meier overall survival curve analysis of patients showed that the five-year survival rate was 49.1% (95% CI, 40.5%-59.7%) in the high-risk group and 76.2% (95% CI, 68.5%-84.9%) in the low-risk group (*p* = 2.7e-6). In addition, the time-dependent ROC curve was plotted to estimate the predictive performance of the risk scoring model. As **Figure 7B** showed the AUC of the prognostic risk assessment model for the three methylation-driven genes (*LAT*, *NFE2L3*, and *HOXD3*) was 0.698. The risk heat map for high and low-risk groups was also plotted in** Figure 7C**.

### Gene expression and methylation patterns of novel DNA methylation-driven genes in GEO datasets

**Figure 8:**
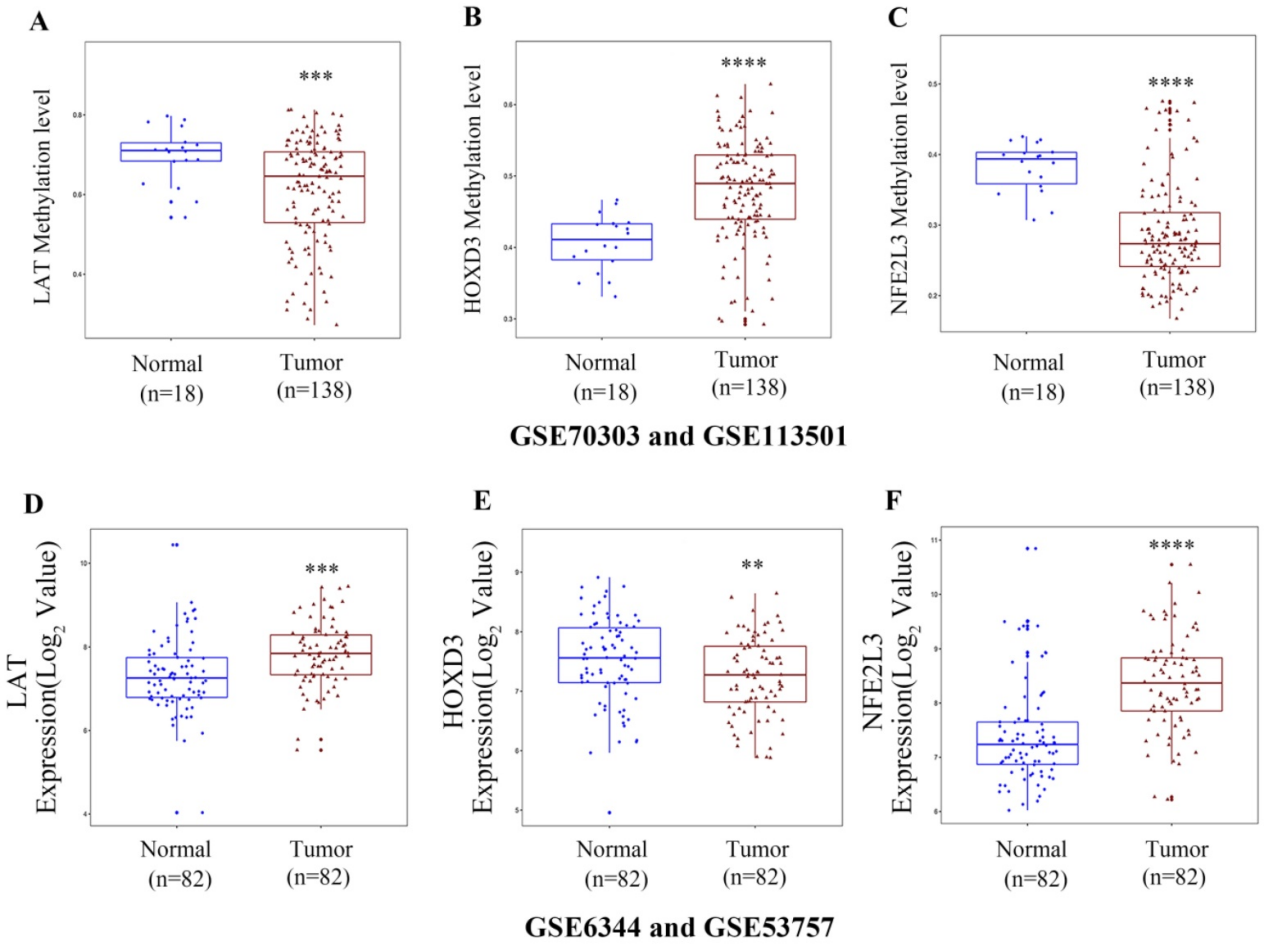
Boxplot showing gene methylation status and expression levels of DNA methylation-driven genes in GEO datasets. (A-C) DNA methylation status of LAT, HOXD3 and NFE2L3 between normal and ccRCC samples. (D-F) gene expression levels of LAT, HOXD3 and NFE2L3 between normal and ccRCC samples. Two-tailed *p* value was determined by unpaired t-test, **, *p* < 0.01, ***, *p* < 0.001, ****, *p* < 0.0001.

To verify the DNA methylation levels and gene expression details of the DNA methylation-driven genes, each of the analysis chose two datasets from GEO website. As **Figure 8A-C** showed that LAT and NFE2L3 exhibited a hypomethylation state in ccRCC cancer group, while HOXD3 exhibited a hypermethylation state in ccRCC cancer group. **Figure 8D-F** showed that all the gene expression degrees of LAT, HOXD3 and NFE2L3 were opposite with DNA methelation levels in ccRCC. LAT and NFE2L3 were upregulated in tumor samples, while HOXD3 was downregulated. These results maintain consistency in the findings of TCGA cohort.

## Discussion

DNA methylation, a critical epigenetic modification carried out by DNA methyltransferases (DNMTs) 22, mainly occurs on the CpG island of genomic DNA 23. More evidence suggest that aberrant methylation profiles are one of the important potential mechanisms that causes severe diseases including cancers 24. In particular, hyper- or hypo-methylation in the promoter regions of genes influences the expressions of respective mRNA, which affects various stages in the tumor development 25. Recent studies showed that DNA methylations had been widely used for the diagnosis, prognosis of different cancer types 26-28.

In this study, 611 RNA-seq samples and 485 methylation samples were selected by TCGA KIRC, in which RNA-seq samples were subjected to univariate Cox proportional-hazards model analysis to screen differential genes that were associated with survival, and methylated samples were screened for differentially methylated genes using MethylMix R package. The intersected genes from previous obtained two gene sets were further proceeded by multivariate Cox analysis. We also determined the relative expressions of novel methylation-driven genes in ccRCC samples, correlation analysis between methylation degrees and gene expressions, GSEA analysis for single methylation-driven gene, joint survival analysis of methylation and expression, survival curve and ROC curve analysis for methylation-driven genes.

After multivariate Cox analysis of intersected genes, five genes including HOXD3, LAT, LUCAT1, HHLA2 and NFE2L3 were carried out for the following correlation analysis (**Figure 3B**). HHLA2, a member of the B7 family, was reported to be involved in the immunosuppression of the tumor microenvironment by modulating human T cell function 29. Chen *et al* group also elucidated that HHLA2 acted as an oncogene in the development of ccRCC 30. LUCAT1, belonging to the type of long noncoding RNA, promoted proliferation of ccRCC cells via the AKT/GSK-3β signaling pathway 31. However, compared to previous studies, expressions of HHLA2 and LUCAT1 were not significantly upregulated and excluded in our next-step study.

Aliases for *LAT* Gene is linker for activation of T-cells family member 1. The protein encoded by this gene was phosphorylated by ZAP-70/Syk protein tyrosine kinases following activation of the T-cell antigen receptor (TCR) signal transduction pathway 32, Interestingly, the role of *LAT* gene in cancers has never been reported. We reported that LAT was hypo-methylated and high expressed with low overall survival rate in ccRCC samples (**Figure 4A, 6A**). GSEA KEGG results showed that LAT may influence the ccRCC progression by regulating ubiquitin mediated proteolysis, renal cell carcinoma, ErbB signaling pathway, tight junction, *et al* (**Figure 5A**).

As for HOXD3, Kron *et al* found that HOXD3 represented high methylation levels in prostate cancer patients 33. Till now, there was no report on HOXD3 to be related with ccRCC progression. In our study, HOXD3 was hyper-methylated and low-expressed with a low overall survival rate in ccRCC samples (**Figure 4B, 6B**). GSEA KEGG and GO BP results showed that HOXD3 may affect the development of ccRCC cells through primary immunodeficiency, cytokine-cytokine receptor interaction, homologous recombination, *et al* (**Figure 5B**).

NFE2L3, a nuclear transcription factor, participates in several biological processes including cell differentiation, inflammation and carcinogenesis 34. The report of Chowdhury *et al*'s group demonstrated that NFE2L3 controlled cancer cell proliferations through a variety of regulatory mechanisms, mainly to induce the expression of UKMK1 to promote cancer cell proliferation 35. Compared with previous studies, we first ever found that NFE2L3 could also drive the development of ccRCC (**Figure 4C**). GSEA result further suggested that NFE2L3 may influence the ccRCC progression by regulating immune activity such as antigen processing and presentation, NOD-like receptor signaling pathway, TOLL-like receptor signaling pathway, regulation of lymphocyte-mediated immunity, adaptive immune response, et al (**Figure 5C**). Hypo-high expression of NFE2L3 may cause the tumor cells escaping from the detection of the immune system by inhibiting the anti-tumor immune effect, and consequently lead to proliferation and metastasis of ccRCC.

Risk model construction, survival curve and ROC curve judgement were then proceeded, and the best risk model was obtained, risk score = LAT (gene expression level) * 0.2693 + HOXD3* (-0.3462) + NFE2L3 * 0.2414; with the median value 0.9140593 of risk score as the cut-off value, the 5-year survival rate in high-risk group of patients (risk score > 0.9140593) was 49.1% (95% CI, 40.5% - 59.7%), and that in low-risk group of patients was 76.2% (95% CI, 68.5% - 84.9%) and AUC was 0.698 as judged by ROC curve, having a good application value. By obtaining the methylation degree of LAT, HOXD3, and NFE2L3 in ccRCC samples, we could calculate whether the patient was in a high-risk or low-risk state and predict his or her five-year survival rate, as a consideration for targeted treatment.

Besides the TCGA data, we verifed the DNA methylation status and gene expression levels of LAT, HOXD3, and NFE2L3 in GEO datasets. Although the study only used computer simulation data and did not validate them with fresh clinical samples, there are enough sample data to suggest that changes in the methylation levels of these three genes can serve as biomarkers for renal clear cell carcinoma. In future research, the specific functions of the selected driver genes in ccRCC progression needed to be investigated.

## Conclusion

Through a series of comprehensive bioinformatics analysis, a total of five DNA methylation-driven genes were screened, among which HOXD3, LAT and NFE2L3 were likely to be closely correlated with the occurrence and development of ccRCC.

Related signaling pathways and biological processes such as “ubiquitin mediated proteolysis”, “primary immunodeficiency” and “antigen processing and presentation” were also enriched. ROC curve judgement for three methylated genes could calculate whether the patient was in a high-risk or low-risk state and predict his or her five-year survival rate. More in-depth studies will be carried out to finally determine the specific role and therapeutic targets of these methylated genes in the development of ccRCC.

## Supplementary Material

Supplementary figures and tables.

Supplementary table S2.

## Figures and Tables

**Table 1:** Univariate Cox regression analysis

Gene	HR	Z	P-value
AIF1L	0.811898605	-3.763264684	0.00016771
PROM2	1.137520592	3.330478104	0.00086697
LUCAT1	1.23291044	4.39232162	1.12E-05
LAT	1.356297295	4.443262185	8.86E-06
HOXD3	0.641660649	-3.908869385	9.27E-05
HHLA2	0.862214241	-4.906090745	9.29E-07
NFE2L3	1.435617688	3.296743917	0.000978126
BHMT	0.872984476	-4.103529362	4.07E-05
SLC17A3	0.900235161	-3.771847759	0.000162043

Abbreviations: Gene, gene symbol; HR, hazard ratio. HR>1: the higher the gene expression was, the higher the risk was. HR<1, the higher the gene expression was, the lower the risk was.

**Table 2:** MethylMix model analysis

Gene ^a^	NormalMean^b^	TumorMean^c^	logFC	pValue	adjustP
LAT	0.69717323	0.59389693	-0.23130461	5.02E-36	1.10E-33
HOXD3	0.32534559	0.42388735	0.38170791	2.58E-42	5.64E-40
NFE2L3	0.36261693	0.26098006	-0.47450671	3.69E-60	8.07E-58

a: Gene symbol; b: Normal group methylation level; c: Cancer group methylation level.

**Table 3:** Correlation analysis between methylation degree and gene expression

Gene	Cor	corPavlue
LAT	5.02E-36	1.10E-33
HOXD3	2.58E-42	5.64E-40
NFE2L3	3.69E-60	8.07E-58

Abbreviations: Gene, Gene symbol; Cor, Correlation.

**Table 4:** CpG site information analysis

Gene	CG ID	UCSC_RefGene_Group	Cor
LAT	cg08331345	TSS1500	-0.413
HOXD3	cg24000528	TSS1500	-0.42
NFE2L3	cg13118545	TSS1500	-0.468

Abbreviations: Gene, Gene symbol; CG ID, CpG site name.

**Table 5:** Multivariate Cox regression analysis with candidate DNA methylation-driven genes

Gene	Coef	Exp(coef)	Se(coef)	Z	P
LAT	0.2693	1.3091	0.0705	3.82	0.00013
HOXD3	-0.3462	0.7074	0.1230	-2.81	0.00488
NFE2L3	0.2414	1.2730	0.1151	2.10	0.03605

Note: Likelihood ratio test=35.78 on 3 df, p=8e-08; n=317, number of evens=103. Coef, beta value; Exp(coef), hazard ratio; Se(coef), standard error; Z= coef/se(coef), the Wald statistic value.

## Abbreviations

ccRCC: clear cell renal cell carcinoma
TCGA: The Cancer Genome Atlas
KEGG: Kyoto Encyclopedia of Genes and Genomes
GSEA: Gene Set Enrichment Analysis
DEGs: Differentially expressed genes
KIRC: Kidney renal clear cell carcinoma
