# Supplementary material for: LAT, HOXD3 and NFE2L3 identified as novel DNA methylation-driven genes and prognostic markers in human clear cell renal cell carcinoma by integrative bioinformatics approaches
Source: J Cancer. 2019 Oct 22;10(26):6726–37. doi: 10.7150/jca.35641 (PMC6856894; doi:10.7150/jca.35641)
Supplement: Supplementary file 1 — Supplementary figures and tables. [file jcav10p6726s1.pdf]

Figure S1

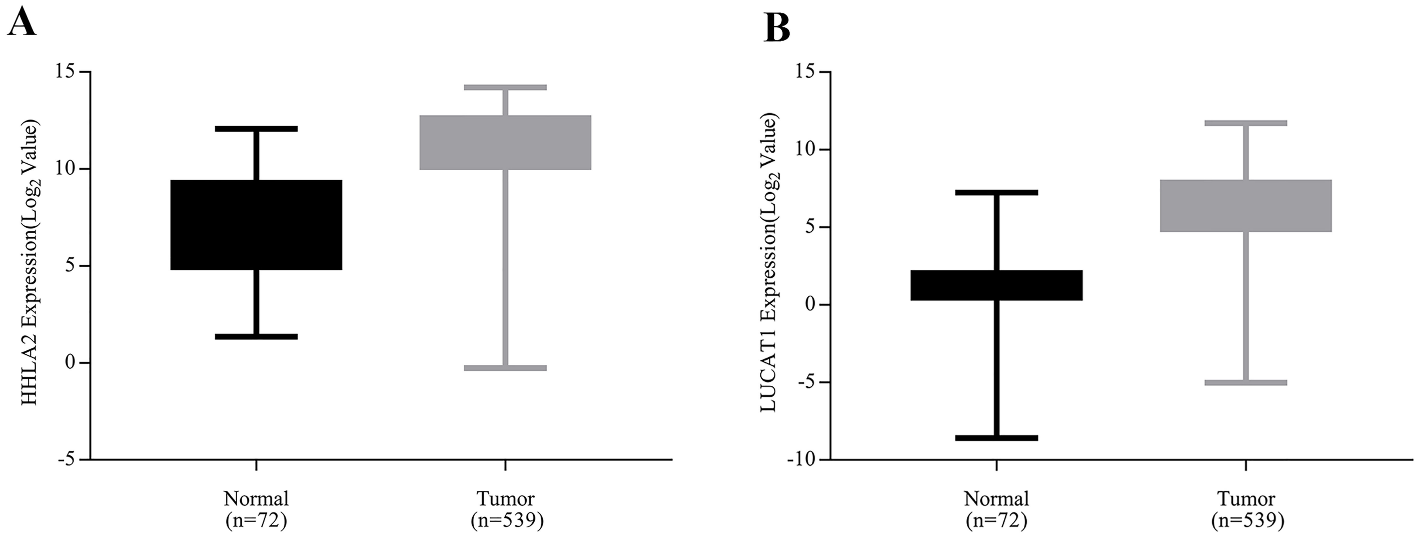
A
B
HHLA2 Expression(Log2 Value)
LUCAT1 Expression(Log2 Value)
15
10
5
0
-5
-10
Normal
(n=72)
Tumor
(n=539)

Figure S2

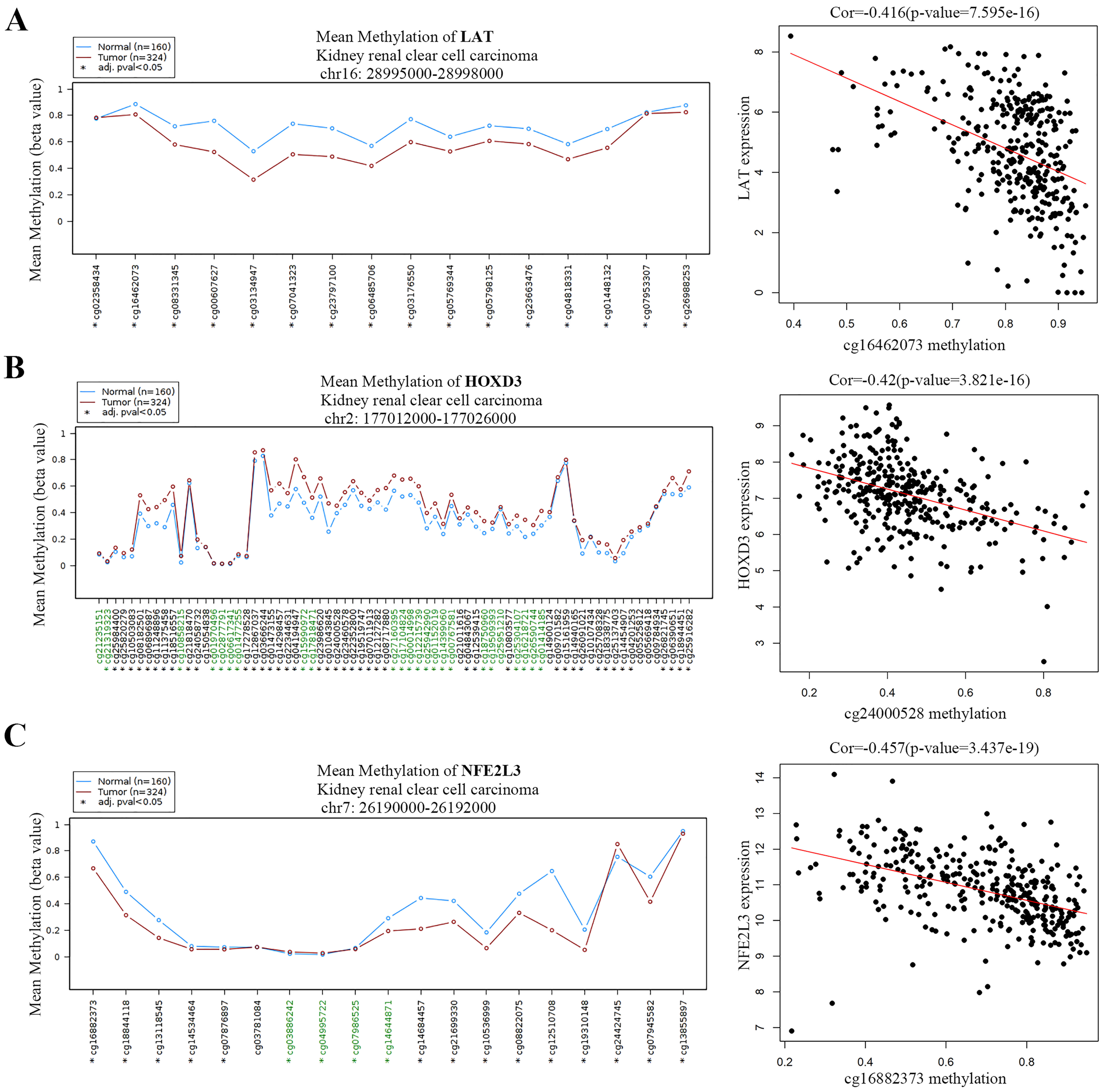

A
Mean Methylation of LAT
Kidney renal clear cell carcinoma
chr16: 28995000-28998000
Normal (n=160)
Tumor (n=324)
* adj. pval<0.05
Mean Methylation (beta value)
cg02358434
cg16462073
cg08331345
cg00607627
cg03134947
cg07041323
cg23797100
cg06485706
cg03176550
cg05769344
cg05798125
cg23663476
cg04818331
cg01448132
cg07953307
cg26988253
Cor=-0.416(p-value=7.595e-16)
LAT expression
cg16462073 methylation
B
Mean Methylation of HOXD3
Kidney renal clear cell carcinoma
chr2: 177012000-177026000
Normal (n=160)
Tumor (n=324)
* adj. pval<0.05
Mean Methylation (beta value)
Cor=-0.42(p-value=3.821e-16)
HOXD3 expression
cg24000528 methylation
C
Mean Methylation of NFE2L3
Kidney renal clear cell carcinoma
chr7: 26190000-26192000
Normal (n=160)
Tumor (n=324)
* adj. pval<0.05
Mean Methylation (beta value)
cg16882373
cg18844118
cg13118545
cg14534464
cg07876897
cg03781084
cg03886242
cg04995722
cg07986525
cg14644871
cg14684457
cg21699330
cg10536999
cg08822075
cg12510708
cg19310148
cg24424745
cg07945582
cg13855897
Cor=-0.457(p-value=3.437e-19)
NFE2L3 expression
cg16882373 methylation

Table S2. The KEGG pathway annotation of candidate DNA methylation-driven gene with the use of GSEA

Gene	Gene sets	NES	FDR q-val
LAT	KEGG_UBIQUITIN_MEDIATED_PROTEOLYSIS	-2.19593	0.0024342
	KEGG_RENAL_CELL_CARCINOMA	-2.17908	0.0012171
	KEGG_ERBB_SIGNALING_PATHWAY	-2.16282	8.11E-04
	KEGG_VASOPRESSIN_REGULATED_WATER_REABSORPTION	-2.14901	6.09E-04
	KEGG_PROSTATE_CANCER	-2.13281	9.18E-04
	KEGG_ENDOCYTOSIS	-2.11924	0.0014711
	KEGG_COLORECTAL_CANCER	-2.11616	0.0012609
	KEGG_ENDOMETRIAL_CANCER	-2.10814	0.0015524
	KEGG_TIGHT_JUNCTION	-2.10151	0.0020144
	KEGG_INSULIN_SIGNALING_PATHWAY	-2.09393	0.0021298
	KEGG_AMINOACYL_TRNA_BIOSYNTHESIS	-2.08709	0.0021191
	KEGG_NEUROTROPHIN_SIGNALING_PATHWAY	-2.07291	0.0027774
	KEGG_REGULATION_OF_ACTIN_CYTOSKELETON	-2.07252	0.0025638
	KEGG_SPHINGOLIPID_METABOLISM	-2.05628	0.0029849
	KEGG_AMYOTROPHIC_LATERAL_SCLEROSIS_ALS	-2.04616	0.0032518
	KEGG_WNT_SIGNALING_PATHWAY	-2.04282	0.0032742
	KEGG_ADHERENS_JUNCTION	-2.04241	0.0032086
	KEGG_THYROID_CANCER	-2.03697	0.0034674
	KEGG_PANCREATIC_CANCER	-2.03547	0.0034144
	KEGG_AXON_GUIDANCE	-2.03312	0.0034044
HOXD3	KEGG_PRIMARY_IMMUNODEFICIENCY	-2.01399	0.0896912
	KEGG_INTESTINAL_IMMUNE_NETWORK_FOR_IGA_PRODUCTION	-1.99349	0.0633269
	KEGG_CYTOKINE_CYTOKINE_RECEPTOR_INTERACTION	-1.90681	0.1036424
	KEGG_HOMOLOGOUS_RECOMBINATION	-1.8854	0.0965935
	KEGG_OTHER_GLYCAN_DEGRADATION	-1.87538	0.0862587
	KEGG_P53_SIGNALING_PATHWAY	-1.81851	0.1228653
	KEGG_AMINO_SUGAR_AND_NUCLEOTIDE_SUGAR_METABOLISM	-1.8068	0.117782
	KEGG_LYSOSOME	-1.77548	0.1374537
	KEGG_HEMATOPOIETIC_CELL_LINEAGE	-1.75654	0.142747
	KEGG_LEISHMANIA_INFECTION	-1.75604	0.1287359
	KEGG_ASTHMA	-1.75089	0.1216946
	KEGG_NOD_LIKE_RECEPTOR_SIGNALING_PATHWAY	-1.73736	0.1246306
	KEGG_TOLL_LIKE_RECEPTOR_SIGNALING_PATHWAY	-1.71207	0.1401099
	KEGG_FC_GAMMA_R_MEDIATED_PHAGOCYTOSIS	-1.68064	0.1615436
	KEGG_GALACTOSE_METABOLISM	-1.65443	0.1444494
	KEGG_ALPHA_LINOLENIC_ACID_METABOLISM	-1.64698	0.1441016
	KEGG_GLYCOSPHINGOLIPID_BIOSYNTHESIS_LACTO_AND_NEOLACTO_SERIES	-1.64465	0.1329486
	KEGG_RIBOFLAVIN_METABOLISM	-1.54373	0.1756841
	KEGG_NICOTINATE_AND_NICOTINAMIDE_METABOLISM	-1.52943	0.1793574
	KEGG_AMYOTROPHIC_LATERAL_SCLEROSIS_ALS	-1.49896	0.178269
NFE2L3	KEGG_INTESTINAL_IMMUNE_NETWORK_FOR_IGA_PRODUCTION	2.31502	9.90E-04
	KEGG_ASTHMA	2.22277	0.0013342
	KEGG_AUTOIMMUNE_THYROID_DISEASE	2.17964	0.0011303
	KEGG_VIRAL_MYOCARDITIS	2.12972	0.0018815
	KEGG_LEISHMANIA_INFECTION	2.12131	0.0016267
	KEGG_TYPE_I_DIABETES_MELLITUS	2.10432	0.0018113
	KEGG_CELL_ADHESION_MOLECULES_CAMS	2.10239	0.0018426
	KEGG_ALLOGRAFT_REJECTION	2.08947	0.0020694
	KEGG_ANTIGEN_PROCESSING_AND_PRESENTATION	2.07726	0.0024161
	KEGG_GRAFT_VERSUS_HOST_DISEASE	2.06089	0.0029941

KEGG_CYTOKINE_CYTOKINE_RECEPTOR_INTERACTION	2.05622	0.0028325
KEGG_CYTOSOLIC_DNA_SENSING_PATHWAY	2.01525	0.005017
KEGG_NATURAL_KILLER_CELL_MEDIATED_CYTOTOXICITY	1.98715	0.0075434
KEGG_NOD_LIKE_RECEPTOR_SIGNALING_PATHWAY	1.98057	0.0080549
KEGG_PRIMARY_IMMUNODEFICIENCY	1.98037	0.0075179
KEGG_HOMOLOGOUS_RECOMBINATION	1.9418	0.0106902
KEGG_TOLL_LIKE_RECEPTOR_SIGNALING_PATHWAY	1.93468	0.0109227
KEGG_MISMATCH_REPAIR	1.87159	0.0213783
KEGG_T_CELL_RECEPTOR_SIGNALING_PATHWAY	1.85554	0.0233218
KEGG_P53_SIGNALING_PATHWAY	1.84839	0.0242923

Abbreviations: Gene, gene symbol; NES, normalized enrichment score; FDR, false discovery rate.

Table S3. The GO functional annotation of candidate DNA methylation-driven gene with the use of GSEA

Gene	Gene sets	NES	FDR q-val
LAT	GO_PROTEIN_TARGETING	-3.339404	0
	GO_TRANSLATIONAL_INITIATION	-3.31827	0
	GO_ESTABLISHMENT_OF_PROTEIN_LOCALIZATION_TO_ORGANELLE	-3.302083	0
	GO_GOLGI_VESICLE_TRANSPORT	-3.287631	0
	GO_AMIDE_BIOSYNTHETIC_PROCESS	-3.259312	0
	GO_PROTEASOMAL_PROTEIN_CATABOLIC_PROCESS	-3.255723	0
	GO_RRNA_METABOLIC_PROCESS	-3.252444	0
	GO_PROTEIN_TARGETING_TO_MEMBRANE	-3.215333	0
	GO_PROTEIN_FOLDING	-3.199061	0
	GO_RIBOSOME_BIOGENESIS	-3.198145	0
	GO_VACUOLAR_TRANSPORT	-3.178438	0
	GO_VIRAL_LIFE_CYCLE	-3.172631	0
	GO_ESTABLISHMENT_OF_PROTEIN_LOCALIZATION_TO_MEMBRANE	-3.168418	0
	GO_PROTEIN_LOCALIZATION_TO_ENDOPLASMIC_RETICULUM	-3.167993	0
	GO_RIBONUCLEOPROTEIN_COMPLEX_BIOGENESIS	-3.153517	0
	GO_ESTABLISHMENT_OF_PROTEIN_LOCALIZATION_TO_ENDOPLASMIC_RETICULUM	-3.152013	0
	GO_ER_TO_GOLGI_VESICLE_MEDIATED_TRANSPORT	-3.142889	0
	GO_POSITIVE_REGULATION_OF_CATABOLIC_PROCESS	-3.135872	0
	GO_REGULATION_OF_CELLULAR_PROTEIN_CATABOLIC_PROCESS	-3.124358	0
	GO_ENDOMEMBRANE_SYSTEM_ORGANIZATION	-3.113897	0
	GO_COFACTOR_METABOLIC_PROCESS	-3.092233	0
	GO_PROTEIN_LOCALIZATION_TO_MEMBRANE	-3.089388	0
	GO_NCRNA_METABOLIC_PROCESS	-3.08425	0
	GO_POSTTRANSCRIPTIONAL_REGULATION_OF_GENE_EXPRESSION	-3.084227	0
	GO_PROTEIN_POLYUBIQUITINATION	-3.082902	0
HOXD3	GO_ADAPTIVE_IMMUNE_RESPONSE	-2.971033	0
	GO_ACTIVATION_OF_IMMUNE_RESPONSE	-2.879108	0
	GO_B_CELL_MEDIATED_IMMUNITY	-2.758972	0
	GO_POSITIVE_REGULATION_OF_INTERFERON_GAMMA_PRODUCTION	-2.754612	0
	GO_B_CELL_RECEPTOR_SIGNALING_PATHWAY	-2.725312	0
	GO_ANTIGEN_RECEPTOR_MEDIATED_SIGNALING_PATHWAY	-2.722894	0
	GO_REGULATION_OF_INTERFERON_GAMMA_PRODUCTION	-2.72241	0
	GO_SISTER_CHROMATID_COHESION	-2.707151	0
	GO_SISTER_CHROMATID_SEGREGATION	-2.654058	0
	GO_ADAPTIVE_IMMUNE_RESPONSE_BASED_ON_SOMATIC_RECOMBINATION_OF_IMMUNE_RECEPTORS_BUILT_FROM_IMMUNOGLOBULIN_SUPERFAMILY_DOMAINS	-2.64444	0
	GO_IMMUNE_EFFECTOR_PROCESS	-2.6421	0
	GO_LYMPHOCYTE_MEDIATED_IMMUNITY	-2.631601	0
	GO_LEUKOCYTE_MEDIATED_IMMUNITY	-2.627825	0
	GO_LYMPHOCYTE_COSTIMULATION	-2.611089	0
	GO_IMMUNE_RESPONSE_REGULATING_CELL_SURFACE_RECEPTOR_SIGNALING_PATHWAY	-2.588453	0
	GO_CHROMOSOME_SEGREGATION	-2.571246	0
	GO_NUCLEAR_CHROMOSOME_SEGREGATION	-2.560045	0
	GO_HUMORAL_IMMUNE_RESPONSE_MEDIATED_BY_CIRCULATING_IMMUNOGLOBULIN	-2.550792	0
	GO_REGULATION_OF_B_CELL_ACTIVATION	-2.542496	0
	GO_POSITIVE_REGULATION_OF_CELL_ACTIVATION	-2.53229	0

	GO_LYMPHOCYTE_ACTIVATION	-2.525446	0
	GO_ACTIVATION_OF_INNATE_IMMUNE_RESPONSE	-2.525433	0
	GO_REGULATION_OF_T_CELL_PROLIFERATION	-2.50515	0
	GO_POSITIVE_REGULATION_OF_T_CELL_PROLIFERATION	-2.503446	0
	GO_PRODUCTION_OF_MOLECULAR_MEDIATOR_OF_IMMUNE_RESPONSE	-2.502215	0
NFE2L3	GO_REGULATION_OF_LYMPHOCYTE_MEDIATED_IMMUNITY	3.051679	0
	GO_ADAPTIVE_IMMUNE_RESPONSE	3.041467	0
	GO_LYMPHOCYTE_COSTIMULATION	3.014854	0
	GO_ACTIVATION_OF_IMMUNE_RESPONSE	2.995625	0
	GO_DEFENSE_RESPONSE_TO_VIRUS	2.982795	0
	GO_REGULATION_OF_INNATE_IMMUNE_RESPONSE	2.97711	0
	GO_POSITIVE_REGULATION_OF_CELL_CELL_ADHESION	2.975723	0
	GO_POSITIVE_REGULATION_OF_CELL_ACTIVATION	2.969666	0
	GO_POSITIVE_REGULATION_OF_LYMPHOCYTE_MEDIATED_IMMUNITY	2.959197	0
	GO_REGULATION_OF_INTERFERON_GAMMA_PRODUCTION	2.958476	0
	GO_REGULATION_OF_HOMOTYPIC_CELL_CELL_ADHESION	2.951035	0
	GO_POSITIVE_REGULATION_OF_INTERFERON_GAMMA_PRODUCTION	2.947577	0
	GO_POSITIVE_REGULATION_OF_T_CELL_PROLIFERATION	2.943495	0
	GO_POSITIVE_REGULATION_OF_INNATE_IMMUNE_RESPONSE	2.933921	0
	GO_REGULATION_OF_LEUKOCYTE_MEDIATED_IMMUNITY	2.927325	0
	GO_INTERFERON_GAMMA_MEDIATED_SIGNALING_PATHWAY	2.918686	0
	GO_LYMPHOCYTE_ACTIVATION	2.916385	0
	GO_LEUKOCYTE_ACTIVATION	2.90934	0
	GO_RESPONSE_TO_VIRUS	2.907774	0
	GO_REGULATION_OF_LEUKOCYTE_PROLIFERATION	2.889759	0
	GO_REGULATION_OF_T_CELL_PROLIFERATION	2.884331	0
	GO_REGULATION_OF_ADAPTIVE_IMMUNE_RESPONSE	2.879126	0
	GO_RESPONSE_TO_INTERFERON_GAMMA	2.878551	0
	GO_POSITIVE_REGULATION_OF_LEUKOCYTE_PROLIFERATION	2.87801	0
	GO_IMMUNE_EFFECTOR_PROCESS	2.876739	0

Abbreviations: Gene, gene symbol; NES, normalized enrichment score; FDR, false discovery rate.
